# Supplementary material for: Human Development Index Is Associated with COVID-19 Case Fatality Rate in Brazil: An Ecological Study
Source: Int J Environ Res Public Health. 2022 Apr 27;19(9):5306. doi: 10.3390/ijerph19095306 (PMC9102208; doi:10.3390/ijerph19095306)
Supplement: Supplementary file 1 [file ijerph-19-05306-s001.zip › ijerph-1676203-supplementary.pdf]

International Journal of
Environmental Research and Public Health

MDPI

Article

Human Development Index Is Associated with COVID-19 Case Fatality Rate in Brazil: An Ecological Study

Camila Vantini Capasso Palamim [1,2,3], Matheus Negri Boschiero [1,2], Felipe Eduardo Valencise [1,2] and Fernando Augusto Lima Marson [1,2,3,*]

1 Laboratory of Cell and Molecular Tumor Biology and Bioactive Compounds, São Francisco University, Avenida São Francisco de Assis, 218, Jardim São José, Bragança Paulista 12916-900, SP, Brazil; cvcpalamim@gmail.com (C.V.C.P.); boschiero.matheus@gmail.com (M.N.B.); felipe.valencise@gmail.com (F.E.V.)

2 Laboratory of Human and Medical Genetics, São Francisco University, Avenida São Francisco de Assis, 218, Jardim São José, Bragança Paulista 12916-900, SP, Brazil

3 Postgraduate Program in Health Science, São Francisco University, Avenida São Francisco de Assis, 218, Jardim São José, Bragança Paulista 12916-900, SP, Brasil

* Correspondence: fernandolimamarson@hotmail.com; Tel.: +55-19-99769-2712

Supplementary Material S1

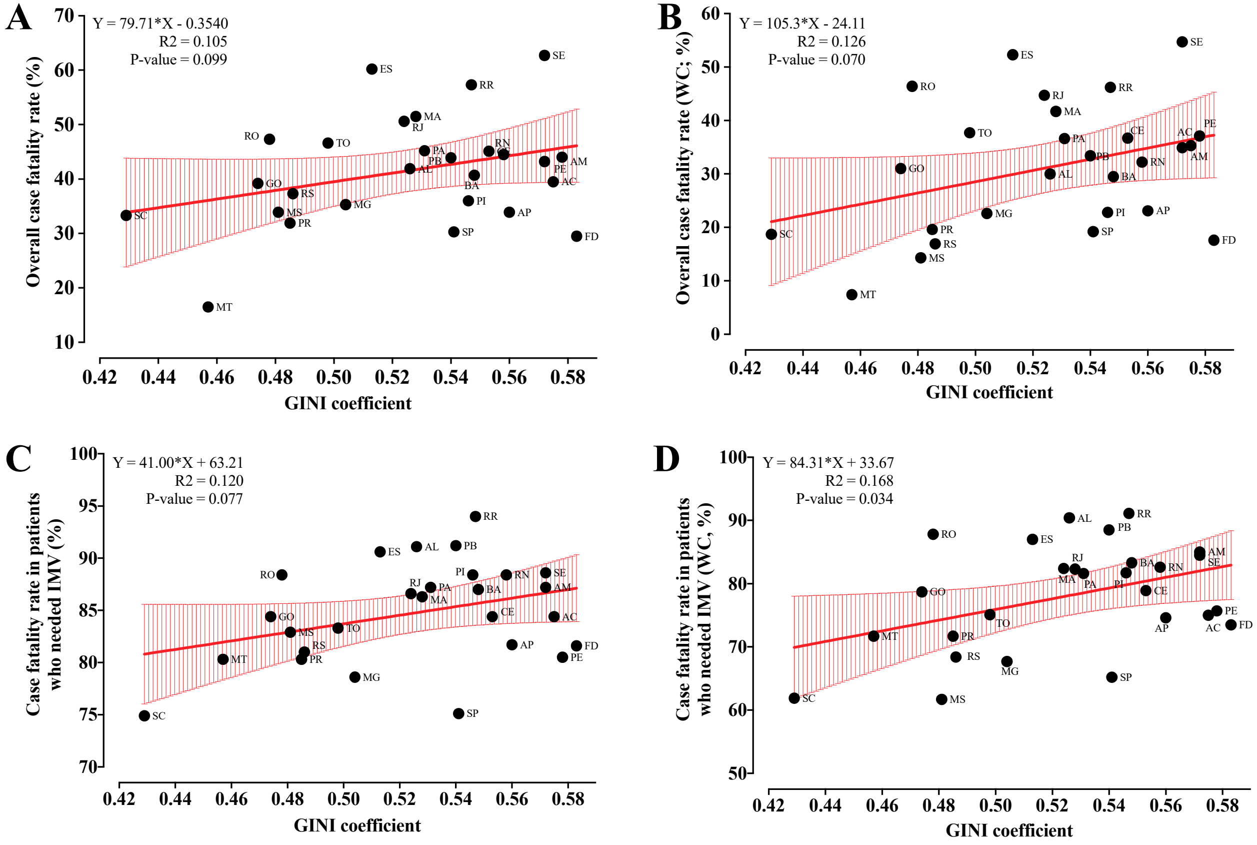

Figure S1. Univariate Regression Analysis between the case fatality rate due to Coronavirus Disease (COVID)-19 and the GINI coefficient of household income per capita, at average prices for the year. **(a)** Overall case fatality rate. **(b)** The overall case fatality rate in COVID-19 individuals without comorbidities (WC). **(c)** The case fatality rate in individuals who received invasive mechanical ventilation (IMV). **(d)** The case fatality rate in individuals who needed IMV and did not have comorbidities. The Y represented the case fatality rate as a dependent marker, and the Y described the GINI coefficient as an independent marker. AC, Acre; AL, Alagoas; AP, Amapá; AM, Amazonas; BA, Bahia; CE, Ceará; ES, Espírito Santo; FD, Federal District; GO, Goiás; MA, Maranhão; MT, Mato Grosso; MS, Mato Grosso do Sul; MG, Minas Gerais; PA, Pará; PB, Paraíba; PR, Paraná; PE, Pernambuco; PI, Piauí; RJ, Rio de Janeiro; RN, Rio Grande do Norte; RS, Rio Grande do Sul; RO; Rondônia; RR, Roraima; SC, Santa Catarina; SP, São Paulo; SE, Sergipe; TO, Tocantins. We obtained the data in OpenDataSUS [1] and from the Brazilian Institute of Geography and Statistics (IBGE) website (Instituto Brasileiro de Geografia e Estatística, in Portuguese) [2].

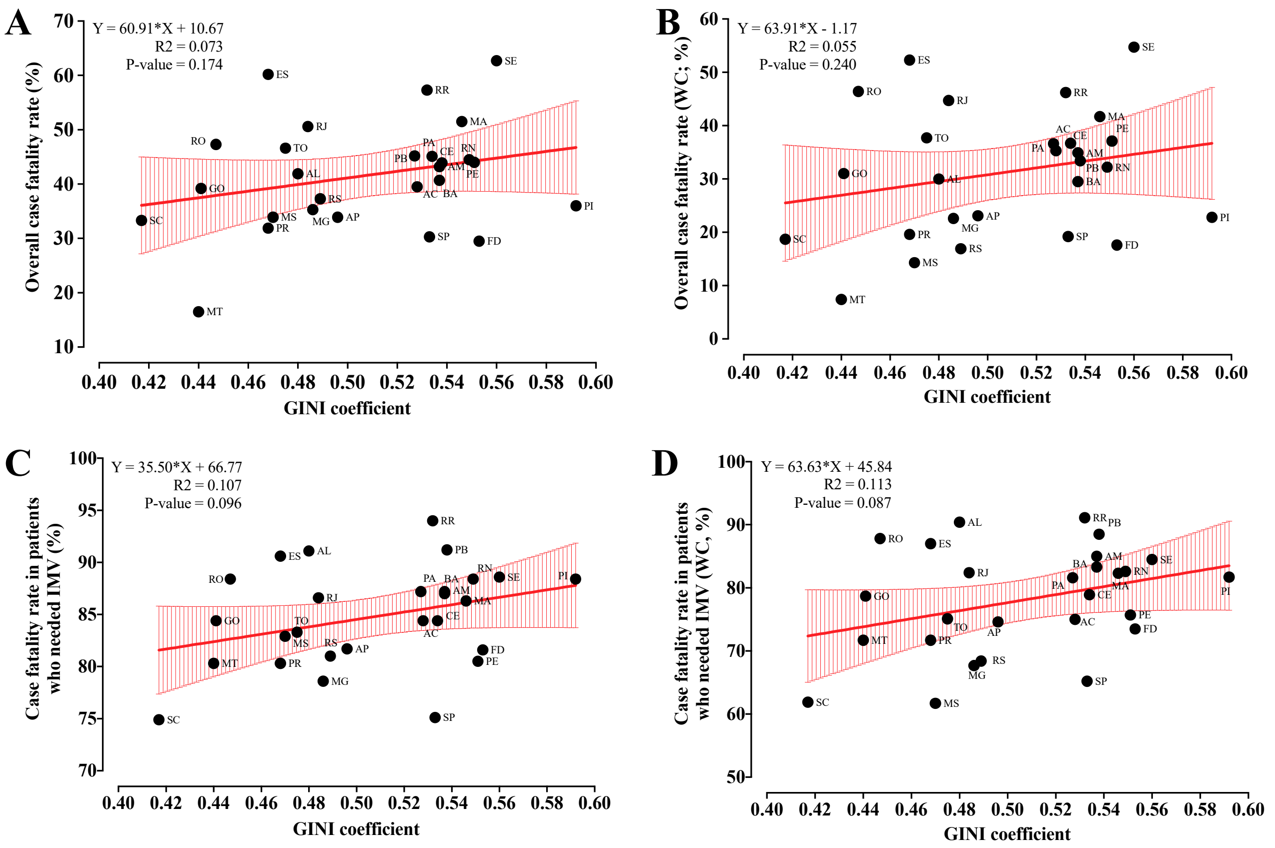

Figure S2. Univariate Regression Analysis between the case fatality rate due to Coronavirus Disease (COVID)-19 and the GINI coefficient of the average real monthly income of people aged 14 and over, actually received in the reference month, for all jobs, at average prices for the year. **(a)** Overall case fatality rate. **(b)** The overall case fatality rate in COVID-19 individuals without comorbidities (WC). **(c)** The case fatality rate in individuals who received invasive mechanical ventilation (IMV). **(d)** The case fatality rate in individuals who needed IMV and did not have comorbidities. The Y represented the case fatality rate as a dependent marker, and the Y described the GINI coefficient as an independent marker. AC, Acre; AL, Alagoas; AP, Amapá; AM, Amazonas; BA, Bahia; CE, Ceará; ES, Espírito Santo; FD, Federal District; GO, Goiás; MA, Maranhão; MT, Mato Grosso; MS, Mato Grosso do Sul; MG, Minas Gerais; PA, Pará; PB, Paraíba; PR, Paraná; PE, Pernambuco; PI, Piauí; RJ, Rio de Janeiro; RN, Rio Grande do Norte; RS, Rio Grande do Sul; RO; Rondônia; RR, Roraima; SC, Santa Catarina; SP, São Paulo; SE, Sergipe; TO, Tocantins. We obtained the data in OpenDataSUS [1] and from the Brazilian Institute of Geography and Statistics (IBGE) website (Instituto Brasileiro de Geografia e Estatística, in Portuguese) [2].

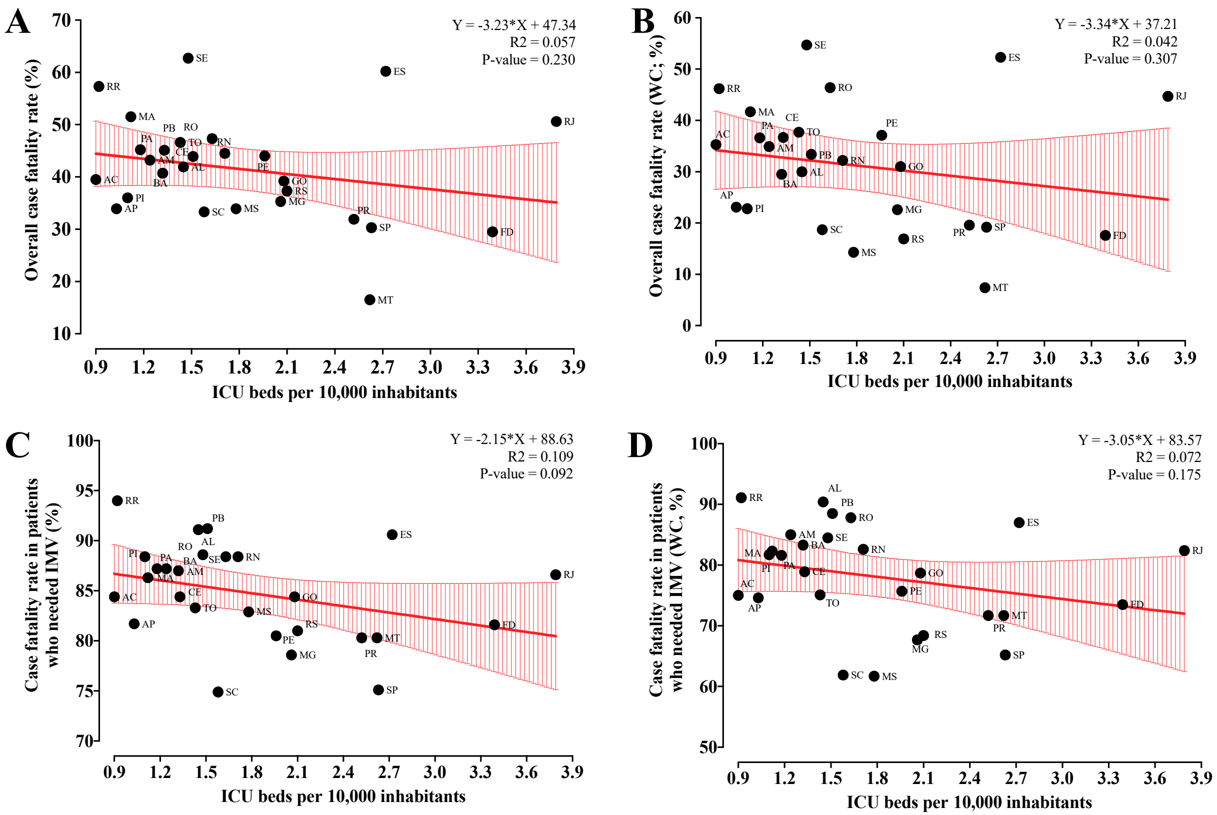

Figure S3. Univariate Regression Analysis between the case fatality rate due to Coronavirus Disease (COVID)-19 and the intensive care unit (ICU) beds per 10,000 inhabitants. **(a)** Overall case fatality rate. **(b)** The overall case fatality rate in COVID-19 individuals without comorbidities (WC). **(c)** The case fatality rate in individuals who received invasive mechanical ventilation (IMV). **(d)** The case fatality rate in individuals who needed IMV and did not have comorbidities. The Y represented the case fatality rate as a dependent marker, and the Y described the ICU beds per 10,000 inhabitants as an independent marker. AC, Acre; AL, Alagoas; AP, Amapá; AM, Amazonas; BA, Bahia; CE, Ceará; ES, Espírito Santo; FD, Federal District; GO, Goiás; MA, Maranhão; MT, Mato Grosso; MS, Mato Grosso do Sul; MG, Minas Gerais; PA, Pará; PB, Paraíba; PR, Paraná; PE, Pernambuco; PI, Piauí; RJ, Rio de Janeiro; RN, Rio Grande do Norte; RS, Rio Grande do Sul; RO; Rondônia; RR, Roraima; SC, Santa Catarina; SP, São Paulo; SE, Sergipe; TO, Tocantins. We obtained the data in OpenDataSUS [1] and from the Federal Council of Medicine website and Palamim and Marson (2020) [3,4].

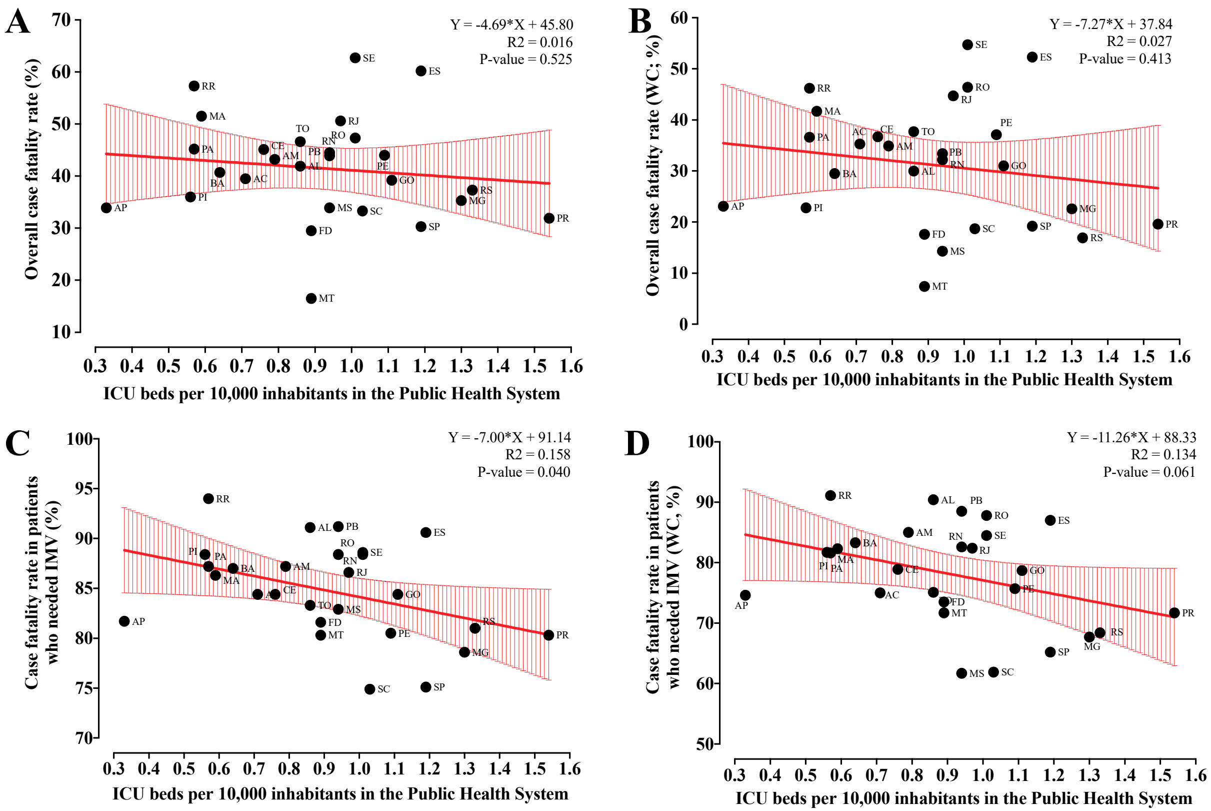

Figure S4. Univariate Regression Analysis between the case fatality rate due to Coronavirus Disease (COVID)-19 and the intensive care unit (ICU) beds per 10,000 inhabitants in the Public Health System. **(a)** Overall case fatality rate. **(b)** The overall case fatality rate in COVID-19 individuals without comorbidities (WC). **(c)** The case fatality rate in individuals who received invasive mechanical ventilation (IMV). **(d)** The case fatality rate in individuals who needed IMV and did not have comorbidities. The Y represented the case fatality rate as a dependent marker, and the Y described the ICU beds per 10,000 inhabitants in the Public Health System as an independent marker. AC, Acre; AL, Alagoas; AP, Amapá; AM, Amazonas; BA, Bahia; CE, Ceará; ES, Espírito Santo; FD, Federal District; GO, Goiás; MA, Maranhão; MT, Mato Grosso; MS, Mato Grosso do Sul; MG, Minas Gerais; PA, Pará; PB, Paraíba; PR, Paraná; PE, Pernambuco; PI, Piauí; RJ, Rio de Janeiro; RN, Rio Grande do Norte; RS, Rio Grande do Sul; RO; Rondônia; RR, Roraima; SC, Santa Catarina; SP, São Paulo; SE, Sergipe; TO, Tocantins. We obtained the data in OpenDataSUS [1] and from the Federal Council of Medicine website and Palamim and Marson (2020) [3,4].

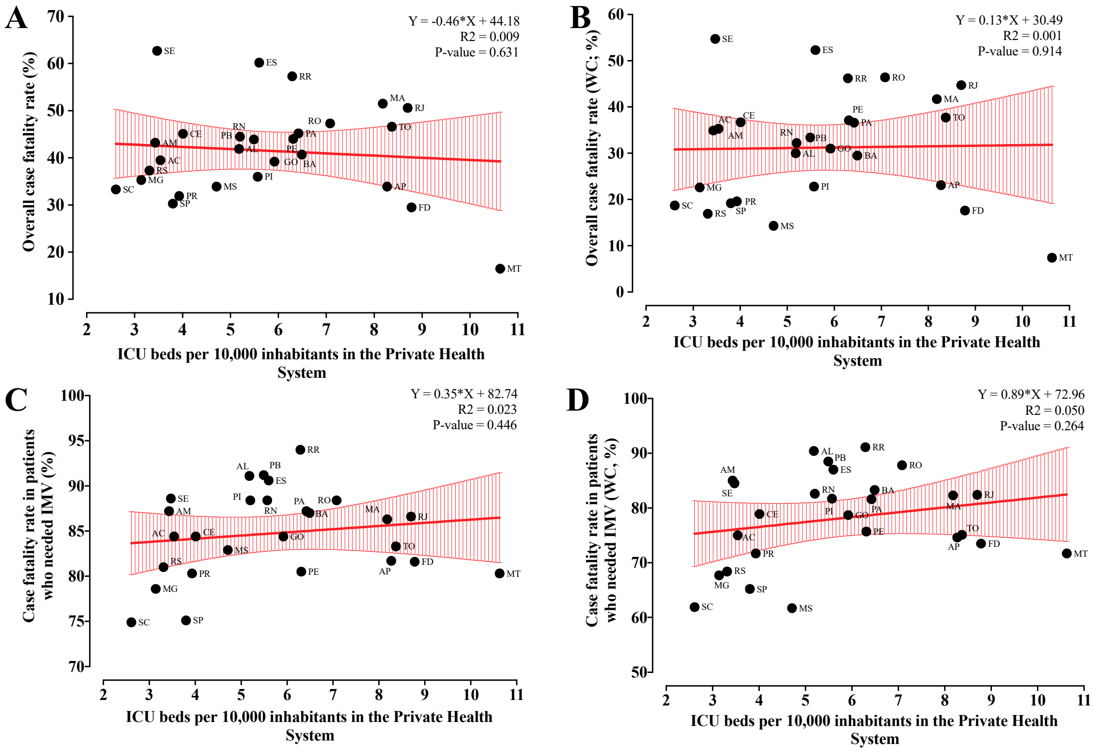

Figure S5. Univariate Regression Analysis between the case fatality rate due to Coronavirus Disease (COVID)-19 and the intensive care unit (ICU) beds per 10,000 inhabitants in the Private Health System. **(a)** Overall case fatality rate. **(b)** The overall case fatality rate in COVID-19 individuals without comorbidities (WC). **(c)** The case fatality rate in individuals who received invasive mechanical ventilation (IMV). **(d)** The case fatality rate in individuals who needed IMV and did not have comorbidities. The Y represented the case fatality rate as a dependent marker, and the Y described the ICU beds per 10,000 inhabitants in the Private Health System as an independent marker. AC, Acre; AL, Alagoas; AP, Amapá; AM, Amazonas; BA, Bahia; CE, Ceará; ES, Espírito Santo; FD, Federal District; GO, Goiás; MA, Maranhão; MT, Mato Grosso; MS, Mato Grosso do Sul; MG, Minas Gerais; PA, Pará; PB, Paraíba; PR, Paraná; PE, Pernambuco; PI, Piauí; RJ, Rio de Janeiro; RN, Rio Grande do Norte; RS, Rio Grande do Sul; RO; Rondônia; RR, Roraima; SC, Santa Catarina; SP, São Paulo; SE, Sergipe; TO, Tocantins. We obtained the data in OpenDataSUS [1] and from the Federal Council of Medicine website and Palamim and Marson (2020) [4].

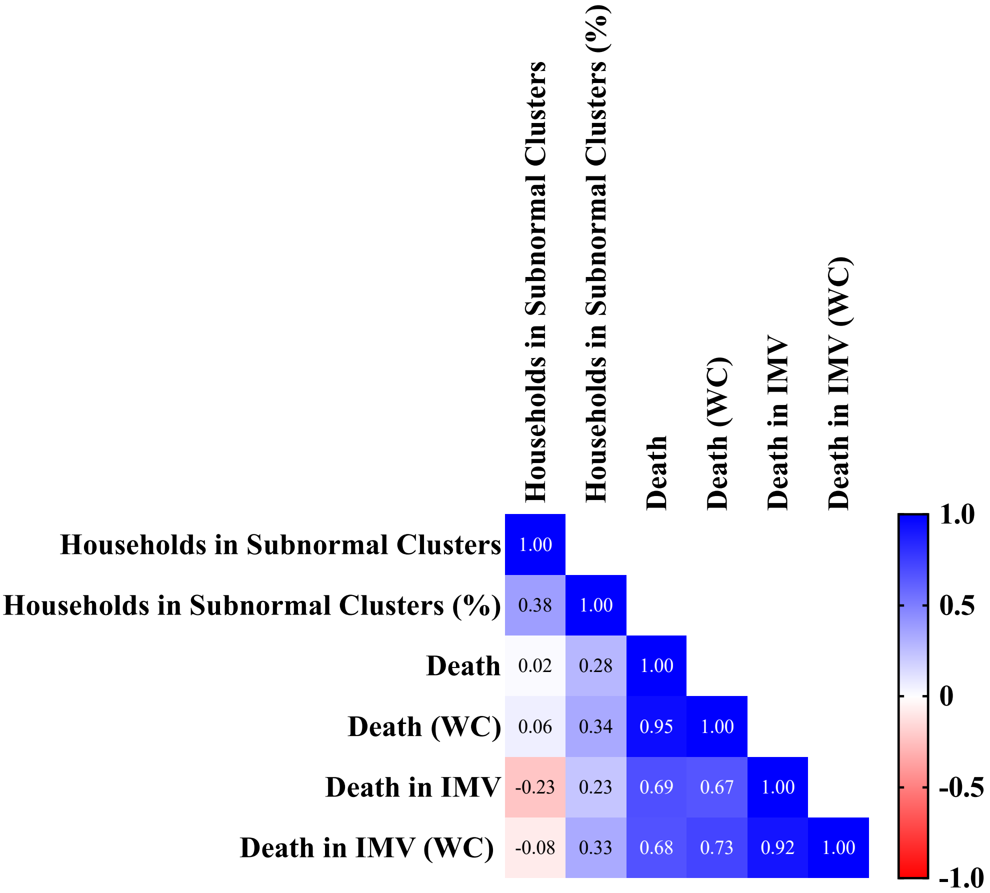

Figure S6. Pearson Correlation Matrix between GINI coefficient and case fatality rate according to the presence of comorbidities and the number/percentage of occupied households in subnormal clusters. We presented the Pearson Correlation Matrix to compare the number/percentage of occupied households in subnormal clusters with the case fatality rate for overall COVID-19 individuals and COVID-19 individuals without comorbidities. We considered the following categorization for the Pearson correlation test: (very high positive/negative correlation) 0.9 to 1.0; (high positive/negative correlation) 0.7 to 0.9; (moderate positive/negative correlation) 0.5 to 0.7; (low positive/negative correlation) 0.30 to 0.50; (negligible correlation) 0.00 to 0.30. We presented an alpha error of 0.05 in all statistical analyses. We presented the case fatality rate as a percentage. WC, without comorbidities; IMV, invasive mechanical ventilation. We obtained the data in OpenDataSUS [1] and from IBGE [2].

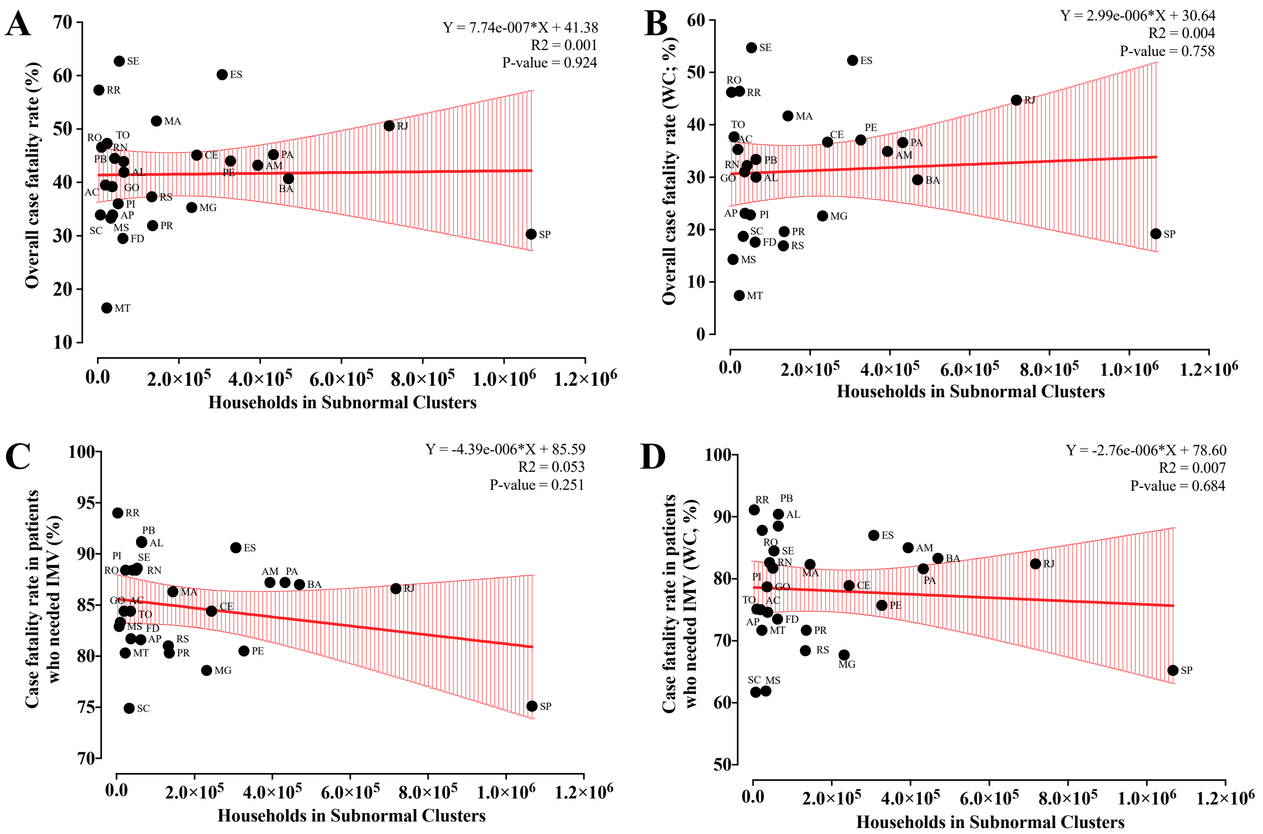

Figure S7. Univariate Regression Analysis between the case fatality rate due to Coronavirus Disease (COVID)-19 and the number of occupied households in subnormal clusters. **(a)** Overall case fatality rate. **(b)** The overall case fatality rate in COVID-19 individuals without comorbidities (WC). **(c)** The case fatality rate in individuals who received invasive mechanical ventilation (IMV). **(d)** The case fatality rate in individuals who needed IMV and did not have comorbidities. The Y represented the case fatality rate as a dependent marker, and the Y described the number of occupied households in subnormal clusters as an independent marker. AC, Acre; AL, Alagoas; AP, Amapá; AM, Amazonas; BA, Bahia; CE, Ceará; ES, Espírito Santo; FD, Federal District; GO, Goiás; MA, Maranhão; MT, Mato Grosso; MS, Mato Grosso do Sul; MG, Minas Gerais; PA, Pará; PB, Paraíba; PR, Paraná; PE, Pernambuco; PI, Piauí; RJ, Rio de Janeiro; RN, Rio Grande do Norte; RS, Rio Grande do Sul; RO; Rondônia; RR, Roraima; SC, Santa Catarina; SP, São Paulo; SE, Sergipe; TO, Tocantins. We obtained the data in OpenDataSUS [1] and from IBGE [2].

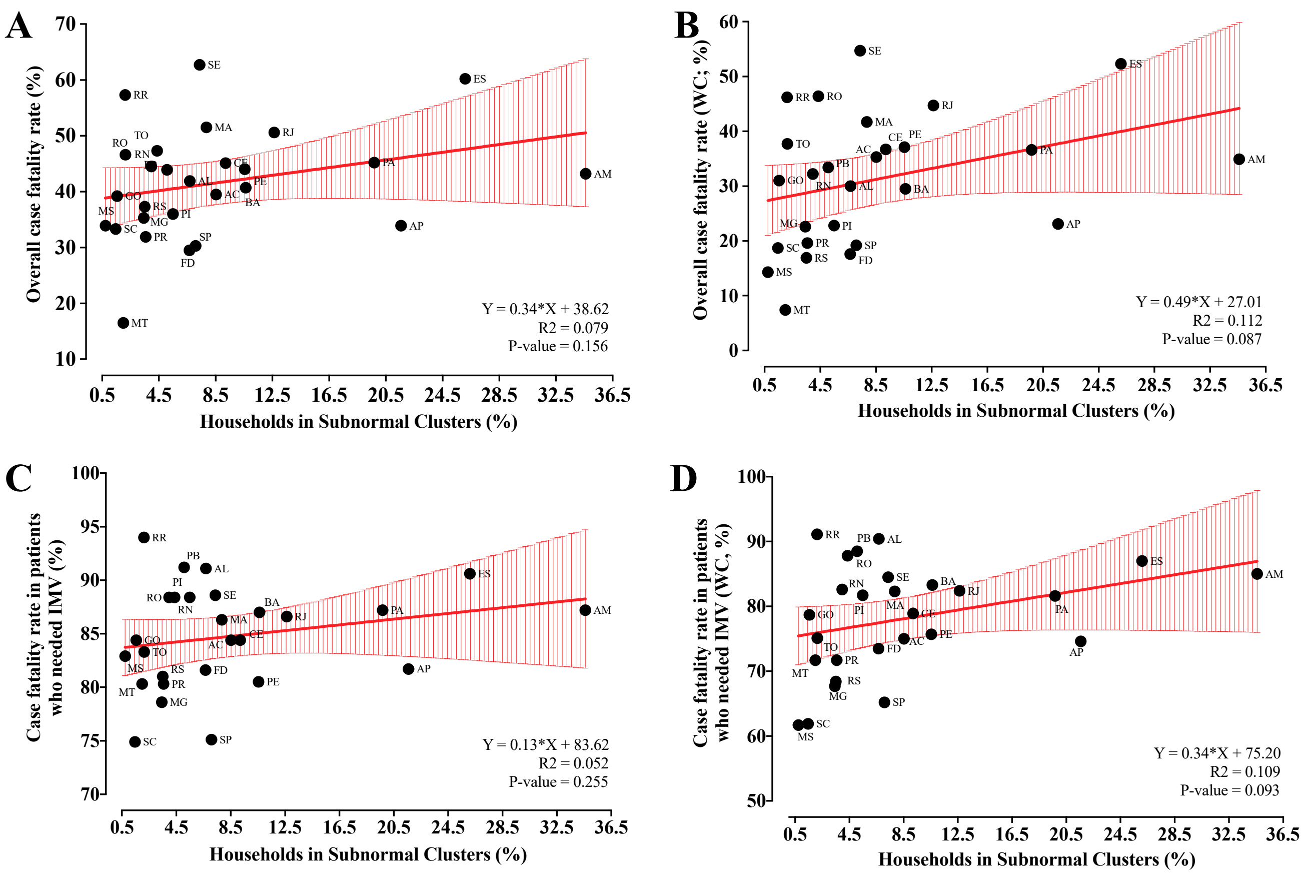

Figure S8. Univariate Regression Analysis between the case fatality rate due to Coronavirus Disease (COVID)-19 and the percentage of occupied households in subnormal clusters. **(a)** Overall case fatality rate. **(b)** The overall case fatality rate in COVID-19 individuals without comorbidities (WC). **(c)** The case fatality rate in individuals who received invasive mechanical ventilation (IMV). **(d)** The case fatality rate in individuals who needed IMV and did not have comorbidities. The Y represented the case fatality rate as a dependent marker, and the Y described the percentage of occupied households in subnormal clusters as an independent marker. AC, Acre; AL, Alagoas; AP, Amapá; AM, Amazonas; BA, Bahia; CE, Ceará; ES, Espírito Santo; FD, Federal District; GO, Goiás; MA, Maranhão; MT, Mato Grosso; MS, Mato Grosso do Sul; MG, Minas Gerais; PA, Pará; PB, Paraíba; PR, Paraná; PE, Pernambuco; PI, Piauí; RJ, Rio de Janeiro; RN, Rio Grande do Norte; RS, Rio Grande do Sul; RO; Rondônia; RR, Roraima; SC, Santa Catarina; SP, São Paulo; SE, Sergipe; TO, Tocantins. We obtained the data in OpenDataSUS [1] and from IBGE [2].

Supplementary Table S1. Description of intensive care unit (ICU) beds* in Brazil according to the States and Federal District.

States	ICU Beds	ICU Beds at the Public Health System	ICU Beds in Private Health System
Acre	75	59	16
Alagoas	491	292	199
Amapá	82	26	56
Amazonas	502	321	181
Bahia	2029	988	1041
Ceará	1201	690	511
Federal District	1031	270	761
Espírito Santo	1091	478	613
Goiás	1409	751	658
Maranhão	787	410	377
Mato Grosso	877	297	580
Mato Grosso do Sul	484	254	230
Minas Gerais	4341	2742	1599
Pará	984	474	510
Paraíba	608	378	230
Paraná	2858	1748	1110
Pernambuco	1861	1034	827
Piauí	353	179	174
Rio de Janeiro	6341	1626	4715
Rio Grande do Norte	601	330	271
Rio Grande do Sul	2375	1506	868
Rondônia	294	183	111
Roraima	48	30	18
Santa Catarina	1108	718	390
São Paulo	11,863	5358	6505
Sergipe	339	230	109
Tocantins	221	134	87

*, The ICUs beds distribution was obtained on the Federal Council of Medicine website and Palamim and Marson (2020) [3,4].

Supplementary Material S2

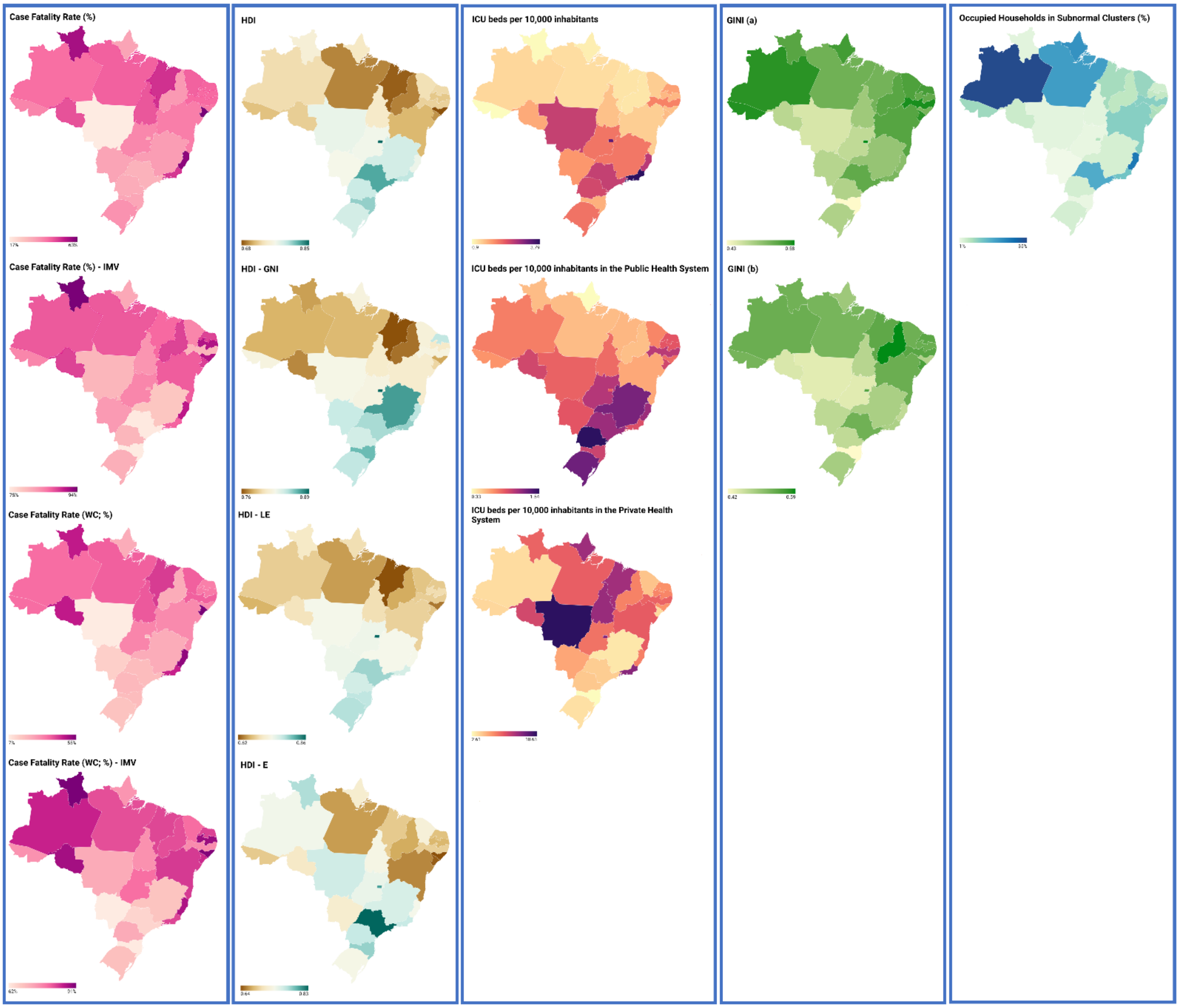

Supplementary Material S2–Figures Legends. Distribution of the features evaluated in the study according to the Brazillian States and Federal District. We presented the information for (i) case fatality rate (%, percentage) according to the need for invasive mechanical ventilation and the presence of comorbidities; (ii) Human Developmental Index (HDI) [including the indicators for the educational level (HDI-E), life expectancy (HDI-LE), and gross national income per capita (HDI-GNI)]; (iii) number of intensive care units (ICU) beds per 10,000 inhabitants – overall, in the Public Health System, and in the Private Health System. The proportion of ICU beds in the Private Health System represents only the number of ICU beds among individuals with access to the Private Health System. In the Public Health System, we consider all Brazillian individuals. (iv) [a], GINI index of household income per capita, at average prices for the year; [b], GINI index of the average real monthly income of people aged 14 and over, actually received in the reference month, for all jobs, at average prices for the year; and (v) relative number (%) of occupied households in subnormal clusters. The case fatality rate was obtained in OpenDataSUS [1] the GINI index was obtained on the Brazilian Institute of Geography and Statistics (IBGE) website (Instituto Brasileiro de Geografia e Estatística, in Portuguese) [2], and the calculation was done in 2017; the number of occupied households in Subnormal Clusters was obtained on the IBGE [2], and the calculation was done in 2019; the ICUs beds distribution was obtained on the Federal Council of Medicine website and Palamim and Marson (2020) [3,4]; the HDI was obtained on the AtlasBR [5] and the calculation was done in 2017.

References

1. Bem Vindo-OPENDATASUS. Available online: https://opendatasus.saude.gov.br/ (accessed on 21 April 2022).
2. IBGE | Portal Do IBGE | IBGE Available online: https://www.ibge.gov.br/ (accessed on 22 January 2022).
3. Available online: https://portal.cfm.org.br/ (accessed on 22 January 2022).
4. Palamim, C.V.C.; Marson, F.A.L. COVID-19—The Availability of ICU Beds in Brazil during the Onset of Pandemic. *Ann. Glob. Health* **2020**, *86*, 100. https://doi.org/10.5334/aogh.3025.
5. Atlas Brasil. Available online: http://www.atlasbrasil.org.br/ranking (accessed on 5 June 2021).
